# Neoadjuvant versus definitive chemoradiation in patients with squamous cell carcinoma of the esophagus

**DOI:** 10.1186/s13014-019-1270-8

**Published:** 2019-04-16

**Authors:** Stefan Münch, Steffi U. Pigorsch, Michal Devečka, Hendrik Dapper, Marcus Feith, Helmut Friess, Wilko Weichert, Moritz Jesinghaus, Rickmer Braren, Stephanie E. Combs, Daniel Habermehl

**Affiliations:** 1 Department of Radiation Oncology, Klinikum rechts der Isar, Technical University Munich, Ismaninger Str. 22, 81675 Munich, Germany; 2 German Cancer Consortium (DKTK), Partner Site Munich, Munich, Germany; 3 Department of Surgery, Klinikum rechts der Isar, Technical University Munich, Ismaninger Str. 22, 81675 Munich, Germany; 4 Institute of Pathology, Klinikum rechts der Isar, Technical University Munich, Ismaninger Str. 22, 81675 Munich, Germany; 5 Institute of Radiology, Klinikum rechts der Isar, Technical University Munich, Ismaninger Str. 22, 81675 Munich, Germany; 6 Helmholtz Zentrum München, Institute of Radiation Medicine (IRM), Ingolstädter Landstraße 1, 85764 Oberschleißheim, Germany

**Keywords:** Squamous cell carcinoma of the esophagus, Definitive chemoradiation, Neoadjuvant chemoradiation, Modern radiation techniques

## Abstract

**Background:**

Multimodal treatment with neoadjuvant chemoradiation followed by surgery (nCRT + S) is the treatment of choice for patients with locally advanced or node-positive esophageal squamous cell carcinoma (E-SCC). Those who are unsuitable or who decline surgery can be treated with definitive chemoradiation (dCRT). This study compares the oncologic outcome of nCRT + S and dCRT in E-SCC patients.

**Methods:**

Between 2011 and 2017, 95 patients with E-SCC were scheduled for dCRT or nCRT+ S with IMRT at our department. Patients undergoing dCRT received at least 50 Gy and those undergoing nCRT + S received at least 41.4 Gy. All patients received simultaneous chemotherapy with either carboplatin and paclitaxel or cisplatin and 5-fluoruracil. We retrospectively compared baseline characteristics and oncologic outcome including overall survival (OS), progression-free survival (PFS) and site of failure between both treatment groups.

**Results:**

Patients undergoing dCRT were less likely to have clinically suspected lymph node metastases (85% vs. 100%, *p* = 0.019) than patients undergoing nCRT + S and had more proximally located tumors (median distance from dental arch to cranial tumor border 20 cm vs. 26 cm, *p* < 0.001). After a median follow up of 25.6 months for surviving patients, no significant differences for OS and PFS were noticed comparing nCRT + S and dCRT. However, the rate of local tumor recurrence was significantly higher in patients treated with dCRT than in those treated with nCRT + S (38% vs. 10%, *p* = 0.002). Within a multivariate Cox regression model, age, tumor location, and tumor grading were the only independent parameters affecting OS and PFS. In addition to that, proximal tumor location was the only parameter independently associated with an increased risk for local treatment failure.

**Conclusion:**

In E-SCC patients treated with either dCRT or nCRT + S, a higher rate of local tumor recurrence was seen in patients treated with dCRT than in patients treated with nCRT + S. There was at least a trend towards an improved OS and PFS in patients undergoing nCRT + S. However, this should be interpreted with caution, because proximal tumor location was the only parameter independently affecting the risk of local tumor recurrence.

## Background

In patients with locally advanced esophageal squamous cell carcinoma (E-SCC) trimodal therapy including neoadjuvant chemoradiation followed by surgery (nCRT + S) demonstrated its potential to improve overall survival (OS) and disease-free survival (DFS) compared to surgery alone [1–3]. Therefore, it has been established as the treatment of choice for patients with non-cervical E-SCC who are suitable for surgery [4]. In contrast, patients with locally advanced E-SCC who decline surgery, are medically inoperable or have unresectable tumors should undergo definitive chemoradiation (dCRT). Because of this, patients undergoing dCRT, show in general more advanced tumors and are in a worse general condition than patients who are treated with nCRT + S. Therefore, a meaningful comparison of both treatment options is difficult. Up to the present there are only two randomized trials comparing dCRT and nCRT + S in patients with esophageal cancer (EC) [5, 6]. The study by Stahl and colleagues, which was terminated early, revealed no significant difference for OS between both treatment groups, while nCRT + S was associated with a significantly increased local progression-free survival [5]. However, all patients in this trial received induction chemotherapy, which is not in line with current treatment recommendations and therefore compromises the interpretation of the results. In accordance with that, a French study also found no difference in OS between surgery or continuation of chemoradiation (CRT) in patients with good response to CRT [6]. Furthermore, there was no significant difference regarding local control rate. One of the major problems of this study is that only patients with good response to CRT were randomized to continuation of chemoradiation or surgery. Therefore, the results are only applicable to a specific subgroup of patients. These results are in line with a recently published retrospective trial by Haefner et al. [7] that compared nCRT + S and dCRT in patients with locally advanced EC. After a median follow-up time of 20.4 months, no significant differences were seen regarding progression-free survival (PFS) and OS between both treatment regimes. In contrast, a recent retrospective cohort study by Barbetta et al. [8] demonstrated an improved OS and DFS after nCRT + S compared to dCRT in patients with thoracic or distal E-SCC. In addition, further retrospective trials also demonstrated an increased survival after nCRT + S in EC patients [9–11]. One major problem with the studies mentioned above is, that most of them included patients with both, adenocarcinoma (AC) and E-SCC. This clearly affects the results because the effect of CRT is higher for patients with E-SCC than for patients with adenocarcinoma [2] and in general these two tumor types – although occurring at the same location - are biology-wise completely different tumor entities. Moreover, many patients were treated with 3-dimensional conformal radiotherapy (3D-CRT). Although no significant differences regarding progression-free survival (PFS) and OS were seen in two retrospective trials comparing 3D-CRT and modern radiation techniques like intensity-modulated radiotherapy (IMRT) for nCRT + S or dCRT in patients with EC [12, 13], the use of modern radiation techniques is at least associated with lower doses to the organs at risk in patients undergoing nCRT + S [12]. Due to the incoherent results of previous trials, our aim is to report the results for patients with only E-SCC, who underwent dCRT or nCRT + S with modern radiation techniques.

## Methods

This study includes 95 patients with E-SCC who were treated with either dCRT or nCRT + S at our department between 2011 and 2017. Inclusion criteria were histologically proven E-SCC, curative treatment approach with either dCRT or nCRT + S, simultaneous chemotherapy, the use of IMRT and at least one follow-up after the end of therapy. Exclusion criteria were distant metastases (M1) at the time of diagnosis, simultaneous radiotherapy or CRT of a second cancer, discontinuation of therapy due to any reason and a total radiation dose < 50 Gray (Gy) for patients undergoing dCRT or < 41.4 Gy for patients undergoing nCRT + S. All patients were staged with ^18^Fludeoxyglucose positron emission tomography combined with computed tomography (^18^FDG-PET/CT) (95%) or computed tomography alone (5%). Whenever possible, endoscopic ultrasound was also used to assess T- and N-stage.

### Treatment

A total of 40 patients were treated with nCRT + S and 55 patients were treated with dCRT. Thereby, dCRT was most commonly used for patients with cervical tumors (47%), patients who refused surgery (27%) and patients who were inoperable due to underlying internistic medical conditions (11%). Additionally, irresectable tumors or high age of patients with reduced general condition were the reasons for choosing dCRT in 7% and 7% of patients, respectively. Treatment planning was based on planning computed tomography (CT) in supine position. All available diagnostic information (esophagogastroduodenoscopy with or without endoscopic ultrasound, ^18^FDG-PET, and CT scans) were used to identify the gross tumor volume (GTV), defined as the macroscopic primary tumor and all putative lymph node metastases. For the planning target volume, a longitudinal safety margin of 4–5 cm and a radial safety margin of 1.5–2 cm were added to the GTV. Due to the long time interval in which patients were included for this analysis and the lack of guidelines regarding elective inclusion of regional lymphatic pathways into the clinical target volume, there was no standardized regional lymphatic coverage policy. In general, the periesophageal and mediastinal lymphatics were at least partially covered by the axial safety margin around the primary tumor. Further coverage of periesophageal and mediastinal lymphatics was done on an individual base, depending on the individual expertise of the treating radiation oncologist. However, additional inclusion of the cervical or abdominal/ coeliac lymphatics was seen in 68% of patients undergoing nCRT + S before 2014 and no patient undergoing nCRT + S after 2014. This difference is caused by the fact, that since 2014 patients were treated analogously to the CROSS-Trial. In patients treated with dCRT, elective nodal irradiation (cervical and/or abdominal/coeliac) was done in 65% of patients. In all patients, irradiation was applied using 6−/or 15 MeV photons delivered with IMRT. Median total radiation dose was 41.4 Gy (range 41.4–45 Gy) for patients treated with nCRT + S and 54 Gy (range 50–64.8 Gy) for patients treated with dCRT, respectively.

In patients undergoing nCRT + S, median radiation dose was 43.2 Gy for patients with cervical tumor location and 41.4 Gy for patients with thoracic or abdominal tumor location. In those undergoing dCRT, median radiation dose was 54 Gy in patients with cervical tumor location, 56 Gy in patients with thoracic tumor location and 55.8 Gy in patients with abdominal tumor location. Thirty-nine patients (98%) who underwent nCRT + S received simultaneous chemotherapy with either carboplatin and paclitaxel or cisplatin and 5-fluoruracil. One patient received simultaneous chemotherapy with only cisplatin. Of patients who underwent dCRT, 48 (87%) received simultaneous chemotherapy with either carboplatin and paclitaxel or cisplatin and 5-fluoruracil (5FU), while one patient (2%) received simultaneous chemotherapy with carboplatin and 5FU, one patient (2%) received carboplatin only, two patients (4%) received cisplatin only and 3 patients (5%) received only fluoropyrimidine-based chemotherapy.

In patients who underwent nCRT + S, the median time interval between neoadjuvant chemoradiation and surgery was 42 days (range 25–86 days) and complete tumor resection was achieved in 97% of patients. Histopathologic tumor response to neoadjuvant treatment was assessed according to the classification published by Becker et al. [14]. Thereby, complete tumor response, less than 10% vital tumor, 10–50% vital tumor and more than > 50% vital tumor was seen in 38%, 43%, 7% and 12% of patients, respectively. Regarding post-surgical morbidity, anastomotic insufficiency was seen in 9% of patients after nCRT + S. No cause of death was seen within the first four weeks after treatment (dCRT or nCRT + S).

### Follow-up

After completion of treatment, all patients were regularly invited to follow-up examinations according to our institutional standard. The first follow-up was scheduled approximately 6–8 week after treatment and included clinical examinations, esophagogastroduodenoscopy, and thoracic computed tomography, thereafter in 3-months intervals or as needed clinically.

### Statistics

Comparison of nominally scaled baseline parameters was done using chi-square test. For ordinally scaled parameters, the Fishers-exact-test was used to compare treatment groups, while Mann-Whitney-U-test was used for interval scaled variables. OS was defined as time between the beginning of treatment and death. Patients lost to follow-up were censored. PFS was defined as the period of time between beginning of treatment and any proven tumor recurrence or death for any reason. Overall survival and progression-free survival where compared using the log-rank test. To analyze the effect of different parameters on OS, PFS and local, regional or distant tumor recurrence we also performed univariate and multivariate Cox regression analyses. All statistical tests were conducted in an exploratory manner on two-sided 5% significance levels using the software *SPSS Statistics 18 version 18.0.0* (IBM SPSS Statistics, Armonk, U. S.).

## Results

### Baseline characteristics

Patients’ baseline clinical data and tumor parameters can be seen in Table 1. Within the dCRT group, patients were slightly older (68 years vs. 65 years) and the rate of male patients (73% vs. 55%) was higher than in the group of patients treated with nCRT + S, but these differences were not statistically significant. In both treatment groups most patients had T3 tumors with moderate or poor tumor cell differentiation (G2/G3). In addition, median tumor length was 5 cm in both groups. The rate of clinically suspected pretherapeutic lymph node metastases was higher in patients undergoing nCRT + S than in patients undergoing dCRT (100% vs. 85%, *p* = 0.019). Tumor location was classified according to the position of the tumor as it was seen in the esophagogastroduodenoscopy (EGD). If the oral tumor margin was seen within the first 3 cm of the esophagus, the tumor was classified as cervical. If the center of the tumor was not more than 3 cm away from the cardia, the tumor was classified as an abdominal tumor. All other tumors were classified as thoracic. While there was a comparable rate of patients with abdominal tumor location in both treatment groups, a higher rate of cervical tumor location (47% vs. 10%) was seen in patients treated with dCRT.

**Table 1:** Patients’ baseline and tumor parameters

Parameter	nCRT + S (*n* = 40)	dCRT (*n* = 55)	*p*-value
Age, (years)			
Median	65	68	0.079
IQR	56–72	62–74	
Male sex	22 (55%)	40 (73%)	0.085
T-stage (cT)			
Tis	0 (0%)	1 (2%)	0.148
T1	1 (3%)	3 (5%)	
T2	7 (18%)	8 (15%)	
T3	32 (80%)	37 (67%)	
T4	0 (0%)	6 (11%)	
N-stage (cN)			
N+	40 (100%)	47 (85%)	0.019
Grading			
G1	1 (3%)	0 (0%)	0.729
G2	20 (53%)	26 (53%)	
G3	17 (45%)	23 (47%)	
Tumor length, (cm)			
Median	5	5	0.445
IQR	3–7	4–7	
Distance from dental arch to cranial tumor border, (cm)			
Median	26	20	< 0.001
IQR	24–30	17–26	
Tumor location			
Cervical	4 (10%)	26 (47%)	< 0.001
Thoracic	35 (88%)	28 (51%)	
Abdominal	1 (3%)	1 (2%)	
Cumulative RT dose, (Gy)			
Median	41.4	54	< 0.001
IQR	41.4–45	54–59.4	
Complete tumor resection	36 (97%)	–	–

*IQR* inter-quartiles-range, *Gy* Gray, *RT* radiotherapy

### Survival

**Fig. 1:**
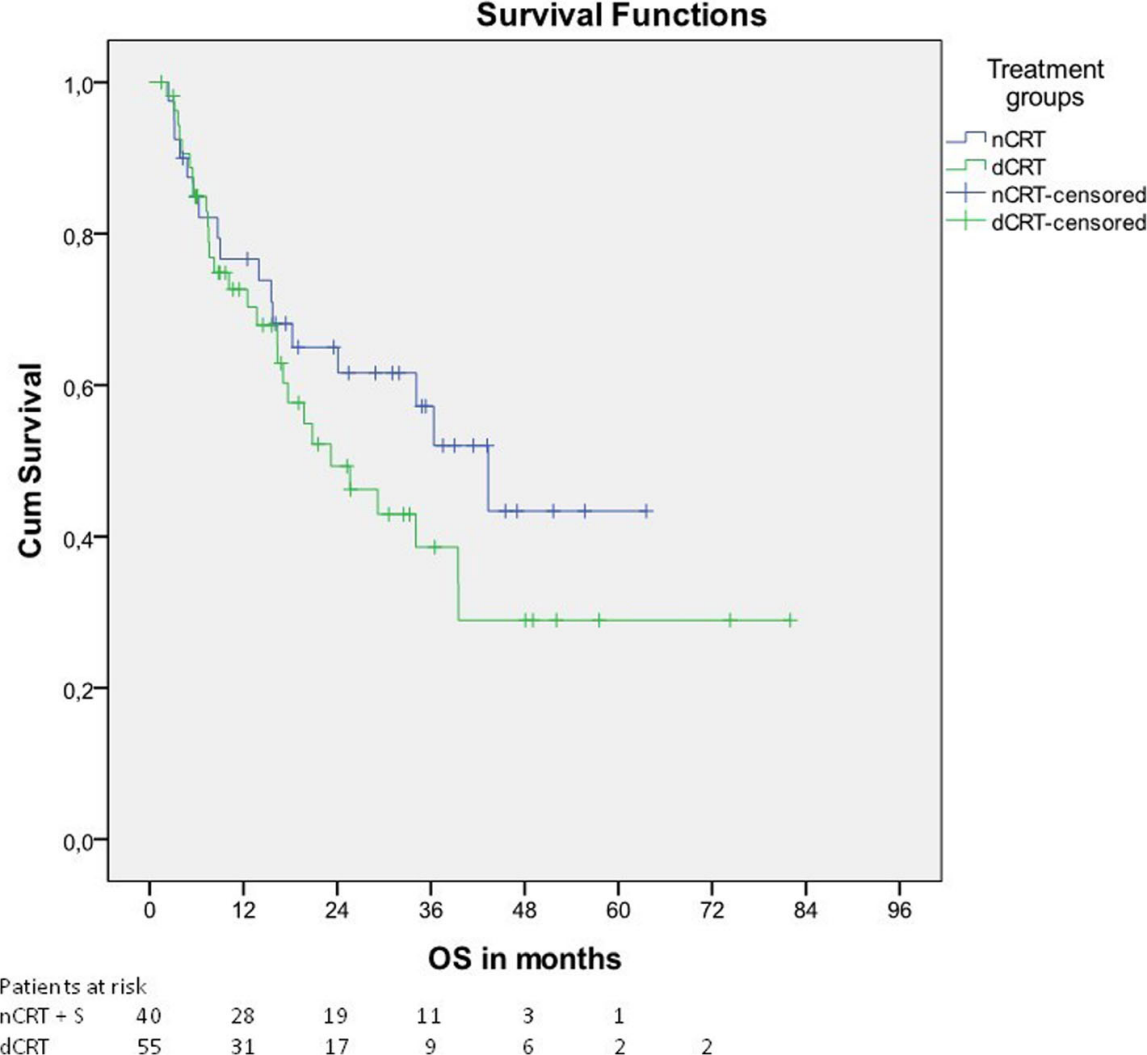
Overall survival

After a median follow-up of 25.6 months for surviving patients, median OS was 43.3 months for patients undergoing nCRT + S and 23.2 months for patients undergoing dCRT (*p* = 0.228). 1y-OS, 2y-OS and 3y-OS was 76.6%, 65.0% and 57.2% (nCRT + S) and 72.6%, 49.3% and 38.6% (dCRT), respectively (Fig. 1).

No significant difference was seen after exclusion of patients with cervical tumor location. After a median follow-up of 28.9 months for surviving patients, median OS was 43.3 months for patients undergoing nCRT + S and 20.8 months for patients undergoing dCRT (*p* = 0.211) (Table 2).

**Table 2:** Oncologic outcome for the whole cohort and for patients with thoracic or abdominal tumor location only

Oncologic Outcome	All patients (*n* = 95)			Patients with thoracic or abdominal tumor location only (*n* = 65)		
	nCRT + S (*n* = 40)	dCRT (*n* = 55)	*p*-value	nCRT + S (*n* = 36)	dCRT (*n* = 29)	*p*-value
Overall survival in months (median)	43.3	23.2	0.228	43.3	20.8	0.211
Progression-free survival in months (median)	18.3	12.7	0.108	19.3	14.0	0.115
Patterns of treatment failure						
Local failure, n (%)	4 (10%)	21 (38%)	0.002	3 (8%)	8 (28%)	0.051
Regional failure, n (%)	9 (23%)	7 (13%)	0.269	7 (19%)	3 (10%)	0.491
Distant failure, n (%)	4 (10%)	9 (16%)	0.547	3 (8%)	4 (14%)	0.691

**Fig. 2:**
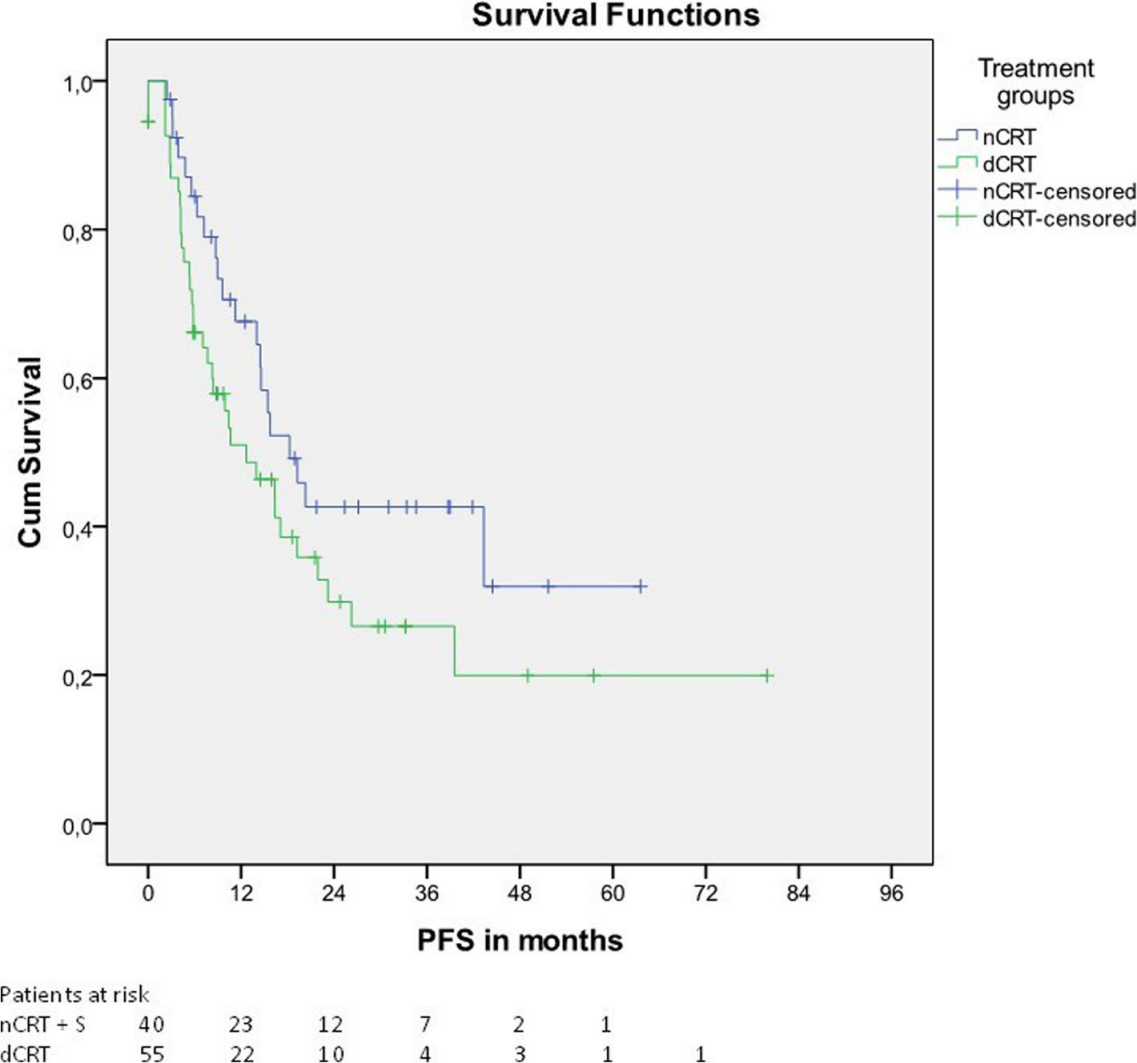
Progression-free survival

Median PFS was 18.3 and 12.7 months in patients treated with nCRT + S and dCRT, respectively (Fig. 2, *p* = 0.108). Corresponding 1y-PFS, 2y-PFS and 3y-PFS were 67.6%, 42.6% and 42.6% in patients undergoing nCRT + S and 51.0%, 29.9% and 26.6% in patients undergoing dCRT.

After exclusion of patients with cervical tumors, median PFS was 19.3 months for patients treated with nCRT + S and 14.0 months for patients treated with dCRT (*p* = 0.231) (Table 2).

### Treatment failure

In summary, local or regional treatment failure was seen in 23% of patients, who were treated with nCRT + S and in 40% of patients, who were treated with dCRT (*p* = 0.081). Table 2 demonstrates patterns of failure for patients treated with dCRT or nCRT + S. While no significant difference was seen for the rate of regional treatment failure (23% (nCRT + S) vs. 13% (dCRT), *p* = 0.269), dCRT was associated with an increased risk of local recurrence (10% (nCRT + S) vs. 38% (dCRT), *p* = 0.002). Distant treatment failure occurred in 10% (nCRT + S) and 16% (dCRT) of patients, respectively (*p* = 0.547). In patients with treatment failure, the first site of recurrence was local/regional, distant or both in 70%, 10% and 20% in patients treated with nCRT + S and 71%, 25% and 4% in patients treated with dCRT (*p* = 0.115). Regarding failure pattern, out-of-field locoregional recurrence was seen in 2 patients (13%). Thereby, one patient underwent nCRT + S without elective nodal irradiation, while the other patient underwent dCRT. Elective nodal irradiation was done at the height of the primary tumor and the longitudinal safety margins in this patient.

When excluding patients with cervical tumor location, there was still a trend towards an increased rate of local tumor recurrence in patients undergoing dCRT (28% vs. 8%; *p* = 0.051). Comparable to the results for the whole cohort, no significant difference was seen for the rate of regional tumor recurrence (10% vs. 19%, *p* = 0.491) or distant treatment failure (14% vs. 8%, *p* = 0.691) between both groups (Table 2).

After treatment failure, 15% of patients received local salvage treatment with either surgery or radio(chemo)therapy, and 59% of patients underwent systemic chemotherapy with palliative intent. 27% of patients received no further specific treatment, but only best-supportive care.

Results of univariate and multivariate Cox regression analyses are demonstrated in Table 3 and Table 4. In the univariate Cox regression model, treatment regimen (nCRT + S vs. dCRT) significantly affected the risk of local recurrence, but not OS or PFS. Grading was the only parameter significantly affecting OS, while tumor location significantly affected PFS and risk of local tumor recurrence. However, in the multivariate model, treatment regime did not significantly affect OS, PFS or local recurrence. Increasing age and good tumor differentiation (G1/2) were associated with worse OS (Hazard ratio (HR) 1.064, 95% confidence interval (95% CI) 1.019–1.110, *p* = 0.005 (age); Hazard ratio (HR) 2.674, 95% confidence interval (95% CI) 1.299–4.503, *p* = 0.008 (G1/2 vs. G3)) and PFS (HR 1.054, 95% CI 1.016–1.093, *p* = 0.005 (age); Hazard ratio (HR) 2.034, 95% confidence interval (95% CI) 1.098–3.768, *p* = 0.024 (G1/2 vs. G3)), while an increasing distance of the dental arch to the cranial tumor border was associated with increased OS (HR 0.939, 95% CI 0.884–0.998, *p* = 0.043), increased PFS (HR 0.925, 95% CI 0.876–0.976, *p* = 0.005) and a lower rate of local recurrence (HR 0.843, 95% CI 0.760–0.935, *p* = 0.001).

**Table 3:** Univariate Cox regression analysis

Parameter	OS HR [95% CI]	*p*-value	PFS HR [95% CI]	*p*-value	LR HR [95% CI]	*p*-value
Treatment Regimen (nCRT + S vs. dCRT)	0.690 [0.377; 1.264]	0.230	0.643 [0.374; 1.107]	0.111	0.199 [0.068; 0.582]	0.003
Age (continuous)	1.025 [0.990; 1.061]	0.160	1.025 [0.994; 1.057]	0.115	1.009 [0.966; 1.053]	0.699
Sex (female vs. male)	0.830 [0.446; 1.545]	0.557	0.807 [0.462; 1.410]	0.451	0.775 [0.334; 1.80]	0.553
Tumor lengths, cm (continuous)	0.996 [0.899; 1.104]	0.946	1.012 [0.925; 1.107]	0.798	1.022 [0.895; 1.167]	0.744
Tumor location (continuous)	0.954 [0.907; 1.004]	0.069	0.940 [0.897; 0.985]	0.010	0.844 [0.773; 0.921]	< 0.001
T-stage (Tis/T1/2 vs. T3/4)	1.295 [0.618; 2.714]	0.493	0.966 [0.485; 1.924]	0.921	1.319 [0.524; 3.316]	0.557
N-stage (N0 vs N1)	1.780 [0.965; 4.560]	0.230	1.167 [0.464; 2.935]	0.743	1.207 [0.284; 5.136]	0.799
Grading (G1/2 vs. G3)	2.059 [1.084; 3.911]	0.027	1.673 [0.961; 2.912]	0.069	2.065 [0.882; 4.837]	0.095

*IQR* inter-quartiles-range, *OS* overall survival, *PFS* progression-free survival, *LR* local recurrence, *HR* Hazard ratio, *CI* Confidence interval

**Table 4:** Multivariate Cox regression analysis

Parameter	OS HR [95% CI]	*p*-value	PFS HR [95% CI]	*p*-value	LR HR [95% CI]	*p*-value
Treatment Regimen (nCRT + S vs. dCRT)	1.453 [0.688; 3.070]	0.327	1.135 [0.592; 2.175]	0.704	0.434 [0.136; 1.382]	0.158
Age (continuous)	1.064 [1.019; 1.110]	0.005	1.054 [1.016; 1.093]	0.005	1.041 [0.984; 1.101]	0.165
Sex (female vs. male)	0.634 [0.316; 1.273]	0.200	0.696 [0.370; 1.311]	0.263	0.763 [0.277; 2.100]	0.601
Tumor lengths, cm (continuous)	1.044 [0.933; 1.168]	0.451	1.033 [0.932; 1.145]	0.541	1.057 [0.912; 1.224]	0.462
Tumor location (continuous)	0.939 [0.884; 0.998]	0.043	0.925 [0.876; 0.976]	0.005	0.843 [0.760; 0.935]	0.001
T-stage (Tis/T1/2 vs. T3/4)	1.434 [0.639; 3.221]	0.383	1.118 [0.527; 2.369]	0.772	1.997 [0.665; 5.993]	0.217
N-stage (N0 vs N1)	1.556 [0.558; 4.337]	0.398	0.821 [0.302; 2.234]	0.699	0.605 [0.135; 2.702]	0.510
Grading (G1/2 vs. G3)	2.674 [1.299; 4.503]	0.008	2.034 [1.098; 3.768]	0.024	2.322 [0.835; 6.453]	0.106

*IQR* inter-quartiles-range, *OS* overall survival, *PFS* progression-free survival, *LR* local recurrence, *HR* Hazard ratio, *CI* Confidence interval

## Discussion

In this analysis, we compared outcome of nCRT + S and dCRT in patients with E-SCC. Thereby, we only included patients who were treated with IMRT, to represent current clinical practice. While no significant differences were seen for OS and PFS, the rate of local tumor recurrence was significantly higher in patients treated with dCRT than in those treated with nCRT + S. However, in a multivariate Cox regression analysis, treatment regime was not independently associated with OS, PFS or rate of local tumor recurrence. Instead, the only parameter independently affecting OS, PFS, and rate of local tumor recurrence was tumor location, while patients’ age and tumor grade were independently associated with OS and PFS.

While our absolute data for OS and PFS are comparable with two other recent studies [7, 8], there are conflicting results in terms of the relative difference between patients treated with nCRT + S and patients treated with dCRT. Haefner and colleagues [7] compared dCRT with nCRT + S in patients with esophageal cancer. In contrast to our study, the authors included patients with AC and patients treated with dCRT received two cycles of adjuvant chemotherapy with cisplatin and 5-fluorouracil. After a median follow-up of 20.4 months, no significant differences were visible for median OS (25.9 months vs. 20.6 months) and PFS (14.9 months vs. 15.6 months). In contrast to that, Barbetta and colleagues [8] reported an improved OS (median OS 2.3 years vs. 3.1 years) and DFS (median DFS 1 year vs. 1.8 years) after nCRT + S. The most obvious difference to the present study is the exclusion of patients with cervical or upper thoracic tumors. In our study, a more proximal tumor location was associated with shorter OS and PFS within multivariate Cox regression analysis. While we found no data evaluating the impact of tumor location after nCRT + S or dCRT in E-SCC patients, proximal tumor location was associated with decreased OS in patients with pT2-3N0M0 carcinoma after surgery alone [15]. As patients with cervical tumor location are typically treated with dCRT [16], and therefore the rate of patients with cervical tumor location is frequently higher within the subgroup of patients treated with dCRT, this might explain the improved OS after nCRT + S in some trials. Interestingly, the only study that excluded patients with cervical or upper thoracic tumor location and performed a propensity score-matched analysis, also reported an improved OS for E-SCC patients undergoing nCRT + S [8]. One can speculate that especially after exclusion of patients with cervical or upper thoracic tumor location the number of patients in most trials would be too low to reveal any significant differences in OS. Another factor which might affect the results is the fact that patients who underwent dCRT in our study had a significantly lower rate of lymph node metastases. This might impact the results since it is well established that lymph node involvement is an important and independent prognostic factor in EC patients [17–19].

In accordance with other studies [5, 8, 10], dCRT was associated with an increased rate of local tumor recurrence in our study. However, Stahl [5] and Liao [10] included patients with different tumor locations including patients with tumors of the cervical esophagus. Based on our results, it is conceivable that the difference in the rate of local tumor recurrence in these studies is biased by differences regarding tumor locations between both cohorts. Although, after excluding patients with cervical tumors, we still recognized a strong trend towards an increased local tumor control after nCRT + S compared to dCRT (*p* = 0.051). In addition, in a recent trial by Barbetta and colleagues [8], which also demonstrated an increased risk of local tumor recurrence after dCRT compared to nCRT + S, this kind of bias was ruled out by performing a 1:1 propensity score-matching approach and excluding patients with tumors of the upper esophagus.

In accordance with the studies by Haefner et al. [7] and Barbetta et al. [8], the most common reason for treatment failure in patients undergoing dCRT was local or regional tumor recurrence. The absolute rate of local and regional recurrences after dCRT in our study was slightly higher than in the study by Haefner and colleagues (local 38% vs. 24%; regional 13% vs. 4%). This difference might be explained by the fact, that patients treated with dCRT were were more likely to have tumors of the cervical esophagus (47% vs. 16%) and were also more likely to have lymph node metastases (85% vs. 77%). Interestingly, our results for local and regional tumor recurrence are comparable to the results by Barbetta and colleagues, despite patients with upper esophageal carcinoma were excluded in their study. The higher rate of regional tumor recurrence in patients undergoing nCRT + S in the study by Barbetta et al., might be explained by the higher rate of patients with lymph node metastases (100% vs. 85%), which is an independent risk factor for both, locoregional and distant recurrence after nCRT + S [20]. Therefore, the difference in terms of the rate of lymph node metastases might also explain the higher rate of regional recurrence after nCRT + S in our study compared to the results by Haefner et al. (23% vs. 5%) [7].

For both, patients treated with nCRT + S and patients treated with dCRT, the rate of distant disease recurrence in our study is remarkably lower than in other recent trials [7, 8]. While the exclusion of patients with AC, who have a significantly higher risk of distant tumor recurrence after dCRT compared to patients with E-SCC [21], might partially explain the higher distant recurrence rate in the study by Haefner and colleagues [7], the reason for the higher rate of distant tumor recurrences in the study by Barbetta et al. [8] remains unclear. However, we have to point out, that the rate of distant recurrences in our study might be slightly underestimated. Due to the retrospective nature of our study, not all patients underwent periodic computed tomography during follow-up. While survival data of patients were completed by contacting the local registration offices, these data provided no further information about tumor recurrence. The use of different chemotherapy regimens (cisplatin/ 5-fluorouracil or carboplatin and paclitaxel) in our study should not affect results, because two retrospective trials did show significant differences regarding oncologic outcome between those two regimens for E-SCC patients undergoing dCRT or nCRT + S [22, 23].

As it was mentioned before, our study has some limitations. One important limitation of this study is its retrospective nature. Also the moderate imbalances regarding tumor parameters between the patient cohorts (rate of lymph node metastases and tumor location) might affect the results and should be kept in mind. However, we also want to mention some strengths of our study. We only included patients with a curative treatment approach and who received complete treatment. That means that patients in the nCRT + S group had to receive at least 41.4 Gy radiation dose and patients within the dCRT group underwent radiation therapy with at least 50 Gy. In addition, all patients received simultaneous chemotherapy.

## Conclusion

In E-SCC patients treated with either dCRT or nCRT + S, a higher rate of local tumor recurrence was seen in patients treated with dCRT than in patients treated with nCRT + S. There was at least a trend towards an improved OS and PFS in patients undergoing nCRT + S. However, this should be interpreted with caution, because proximal tumor location was the only parameter independently affecting the risk of local tumor recurrence.

## Abbreviations

3D-CRT: 3-dimensional conformal radiotherapy
5FU: 5-fluoruracil
AC: Adenocarcinoma
CRT: Chemoradiation
CT: Computed tomography
dCRT: Definitive chemoradiation
DFS: Disease-free survival
EC: Esophageal cancer
E-SCC: Esophageal squamous cell carcinoma
^18^FDG-PET/CT: ^18^Fludeoxyglucose positron emission tomography with computed tomography
GTV: Gross tumor volume
Gy: Gray
IMRT: Intensity-modulated radiotherapy
nCRT + S: Neoadjuvant chemoradiation followed by surgery
OS: Overall survival
PFS: Progression-free survival
PTV: Planning target volume
